# Gene Duplications in the Genomes of Staphylococci and Enterococci

**DOI:** 10.3389/fmolb.2020.00160

**Published:** 2020-07-23

**Authors:** José Francisco Sanchez-Herrero, Manuel Bernabeu, Alejandro Prieto, Mário Hüttener, Antonio Juárez

**Affiliations:** ^1^Department of Genetics, Microbiology and Statistics, University of Barcelona, Barcelona, Spain; ^2^Biodiversity Research Institute (IRBio), University of Barcelona, Barcelona, Spain; ^3^High Content Genomics and Bioinformatics Unit, Germans Trias i Pujol Research Institute (IGTP), Campus Can Ruti, Badalona, Spain; ^4^Institute for Bioengineering of Catalonia, The Barcelona Institute of Science and Technology, Barcelona, Spain

**Keywords:** gene duplication, *Staphylococcus aureus*, *Enterococcus faecium*, *Enterococcus faecalis*, bacterial genomics

## Abstract

Gene duplications are a feature of bacterial genomes. In the present work we analyze the extent of gene duplications in the genomes of three microorganisms that belong to the Firmicutes phylum and that are etiologic agents of several nosocomial infections: *Staphylococcus aureus*, *Enterococcus faecium*, and *Enterococcus faecalis*. In all three groups, there is an irregular distribution of duplications in the genomes of the strains analyzed. Whereas in some of the strains duplications are scarce, hundreds of duplications are present in others. In all three species, mobile DNA accounts for a large percentage of the duplicated genes: phage DNA in *S. aureus*, and plasmid DNA in the enterococci. Duplicates also include core genes. In all three species, a reduced group of genes is duplicated in all strains analyzed. Duplication of the *deoC* and *rpmG* genes is a hallmark of *S. aureus* genomes. Duplication of the gene encoding the PTS IIB subunit is detected in all enterococci genomes. In *E. faecalis* it is remarkable that the genomes of some strains encode duplicates of the *prgB* and *prgU* genes. They belong to the *prgABCU* cluster, which responds to the presence of the peptide pheromone cCF10 by expressing the surface adhesins PrgA, PrgB, and PrgC.

## Introduction

Gene duplication is an event in which one gene gives rise to two genes that cannot be operationally distinguished from each other. The duplicated genes remain in the same genome. Gene duplications are among the oldest and perhaps the most frequent of mutation types (Lynch et al., 2008; Lipinski et al., 2011). A duplicated gene provides a greater chance for natural selection to shape a novel function (Long et al., 2003). Gene duplication occurs both in eukaryotes and prokaryotes, and significantly impact their gene repertoires, generating functional diversity and increasing the genome complexity (Zhang, 2003; Conant and Wolfe, 2008; Serres et al., 2009; Innan and Kondrashov, 2010; Gao et al., 2017). Duplication events are highly relevant from a biological point of view because, whenever cellular growth is restricted, escape from these growth restrictions can occur by duplication events that resolve the selective problem. In turn, novel duplication events may facilitate subsequent genetic change by allowing cells to proliferate, hence increasing the probability for subsequent adaptive mutations to occur either in the amplified genes or in unrelated ones (Andersson and Hughes, 2009).

In the bacterial kingdom, gene duplication has been associated with survival in extreme or fluctuating conditions, including exposure to antimicrobial compounds or growth on poor nutrient sources, and may have a role in the coevolution between host and pathogens (Romero and Palacios, 1997; Riehle et al., 2001; Duvernay et al., 2011; Kondrashov, 2012; Sun et al., 2012; Toussaint et al., 2017). Several examples correlating gene duplication with bacterial adaptation to the environment are available. For instance, when high gene dosage confers selective benefits, bacteria maintain tandem arrays of duplicated genes (as previously reviewed Romero and Palacios, 1997; Andersson and Hughes, 2009). There is a high natural frequency of bacterial gene duplication, which exceeds the rate of spontaneous point mutation by several orders of magnitude (Andersson and Hughes, 2009). Recent studies indicate that more than 20% of cells in a population contain duplications in some genomic region despite the absence of any evident selection for such duplications (Anderson and Roth, 1981; Hooper and Berg, 2003; Treangen and Rocha, 2011; Elliott et al., 2013).

In most studies the presence of gene duplications is restricted to specific genes or genomic regions, and a global view of the impact of gene duplications in the bacterial genomes is missing. *Escherichia coli* is an example. Previous studies in *E. coli* had shown that some genes such as *flu*, which encodes the adhesin Ag43, can be present in several copies in different strains (van der Woude and Henderson, 2008; Elliott et al., 2013; Arun et al., 2016), but until recently the extent of gene duplications in the genomes of the different types of pathogenic *E. coli* has not been available (Bernabeu et al., 2019). Most pathogenic *E. coli* strains harbor between 80 and 100 duplicated genes. Despite the high genomic diversity of *E. coli*, a group of about 25 genes is duplicated in most of the virulent *E. coli* strains, irrespective of the pathotype to which they belong (Bernabeu et al., 2019). Most of those genes code for proteins of unknown function and, as they are absent from the genomes of commensal strains, their gene products likely play a role in virulence.

In the present report we have undertaken a whole-genome analysis of gene duplications in the genomes of some of the most clinically relevant Gram-positive cocci, namely *Staphylococcus aureus, Enterococcus faecium*, and *Enterococcus faecalis*.

*S. aureus and E. faecium* are the Gram-positive representatives of the ESKAPE group, which includes microorganisms that are frequent causes of life-threatening nosocomial infections and display multiple antibiotic resistance phenotypes (Murray, 2000; Naimi et al., 2001; Torell et al., 2005). Staphylococcal and enterococcal bacteremia are prevalent in hospitalized patients, and are associated with significant morbidity and mortality (Bartash and Nori, 2017). The emergence of *S. aureus* strains resistant to many antibiotics, including methicillin-resistance (MRSA), poses a serious threat to human health even in countries with well-developed health surveillance systems. Some *E. faecium* and *E. faecalis* isolates account for about 15% of hospital acquired infections in Europe and the US (Werner et al., 2008; Zarb et al., 2012). *E. faecium* infections are nowadays of major concern because of their multidrug resistance phenotypes, including resistance to vancomycin (VRE) and ampicillin. Strains of *E. faecalis* are commensals of the gut microbiota, but under some circumstances they can be pathogenic. Pathogenic strains of *E. faecalis* are increasingly recognized as serious clinical threats due to both the acquisition of multiple antibiotic resistance determinants and to their capacity to disseminate resistance and virulence features by horizontal gene transfer (HGT) mechanisms (Kao and Kline, 2019). Coinfection of MRSA with VRE can occur, being VRE able to transfer VR to the staphylococci (Kos et al., 2012; McGuinness et al., 2017; Cong et al., 2020).

The genomic analysis performed in this work highlights the importance of some genes in the physiology of staphylococci and enterococci. Some of the identified duplicates likely play a role in virulence and hence can be considered as targets of antimicrobial therapies designed to combat infections caused by these pathogens.

## Materials and Methods

### Bacterial Strains and Data Retrieval

We retrieved and analyzed data (genomic fasta, genbank format file, and the translated coding sequences) of all *S. aureus* (*n* = 473), *E. faecium* (*n* = 133), and *E. faecalis* (*n* = 40) complete assembled genomes from NCBI Refseq (Supplementary Table S1).

### Strategy Used for the Analysis of Duplicates

For each of the strains studied, irrespective of the species, we downloaded the data and analyzed the extent of gene duplications within its genome. Once each strain was analyzed, we summarized the results obtained and generated the corresponding analysis at the level of species. For each of the three species analyzed we selected a reference strain and only for these we created visual representations of their duplicates. Then we analyzed the gene duplications shared with other strains of the same species. We also analyzed further specific characteristics of each of the species analyzed for further interpretation of the data.

### Gene Duplication Within Strain

For the analysis of gene duplications we performed an all-vs.-all BLASTp (Altschul et al., 1990) protein similarity search using the translated coding sequence regions and filtering the results with a similarity cutoff >85%, an alignment length between pairs >85%, bit-score >50, and an *e*-value <10-10. We discarded auto hits and grouped duplicates accordingly.

### Analysis of Inserted Phages

We analyzed the putative insertion of phages in the bacterial genomic sequences by using the PhiSpy tool (Akhter et al., 2012) and the genbank format file (gbk) for each of the strains. We used the appropriate training set for each strain according to their species and additional default parameters.

### Phylogenetic Reconstruction

We generated a phylogenetic and clusterization analysis using all strains for each of the species analyzed. We used Mash (Ondov et al., 2016) and Sourmash (Titus and Irber, 2016) as they extend a dimensionality-reduction technique to include a pairwise mutation distance enabling the efficient clustering of massive sequence collections. We employed the genomic FASTA files downloaded for each strain and default parameters. For each species, the order of the strains in the different tables follows its phylogenetic relationship.

### Exploratory Analysis

We summarized and plotted using R^1^ the duplicated count of groups and genes identified and the number of proteins encoding for transposases, selected within the functional annotation associated. We also analyzed the correlation between the amount of duplicated genes and some variables of interest: phages inserted and duplicated proteins annotated as transposases, hypothetical proteins or proteins of unknown function.

### Duplicate Coordinates Visualization

For the visualization of duplicates in the corresponding genomes we retrieved, for each duplicate, genomic features such as the start and end coordinates and strand harboring the coding sequence [either from the genomic feature format file (gff) or from the genomic features within the fasta sequence header]. By using R package BioCircos (Cui et al., 2016) we created a circular representation of the duplicate coordinates along the main chromosome and plasmid sequences if any.

### Gene Duplications Shared Between Strains

For the analysis of gene duplications shared with other strains of the same species, we selected a single sequence within each duplicated group and we employed BLASTp with filtering parameters as above (similarity >85%, alignment >85%, bit-score >50, and *e*-value <10-10).

### Analysis of the Genes Associated to the Generation of Small Colony Variants (SCV)

The analysis of the presence of SCV-associated genes (*deoC*, *sstD*, *plsY*, and *eap)* was done in several staphylococcal strains both coagulase + (*S. aureus*) and coagulase - (*S. epidermidis*, *S. carnosus*, and *S. xylosus*). We downloaded from NCBI Refseq the complete assembled genomes for *S. epidermidis* (*n* = 29) and any available genome for *S. carnosus* (*n* = 10) and *S. xylosus* (*n* = 57) (Supplementary Table S2). Homology search was done by using BLASTp and the translated coding sequences of these selected genes (Supplementary Table S3) and *Staphylococcus* spp. proteomes. In this case, we used less stringent filtering parameters, a similarity cutoff >50%, following protein homology guidelines (Pearson, 2013), and other parameters as above (alignment >85%, bit-score >50, and *e*-value <10–10).

### Analysis of Methicillin and Vancomycin Resistance

Methicillin and vancomycin resistance determinants were searched for each strain by using the Comprehensive Antibiotic Resistance Database (CARD) (Alcock et al., 2020). They were clustered in operons as previously reported (Courvalin, 2006; Shore and Coleman, 2013). We also used BLASTp to identify the presence of each determinant (similarity >80%, alignment >85%, bit-score >50, and *e*-value <10-10) and manually curated the results. The presence of *mecA* or *mecC* genes in *S. aureus* (CARD IDs ARO: 3000617 and 3001209, respectively) was considered to confer the methicillin resistance phenotype (MR) and the presence in *Enterococcus* of *vanA*, *vanB*, and/or *vanG* genes (CARD IDs ARO: 3000010, 3000013, and 3002909, respectively) was considered to confer the vancomycin resistance phenotype (VRE).

### Code Availability

The bioinformatics scripts employed for the analysis were deposited and are available at the github website: https://github.com/molevol-ub/BacterialDuplicates.

## Results

### Gene Duplications in *Staphylococcus* and *Enterococcus* Genomes

**FIGURE 1:**
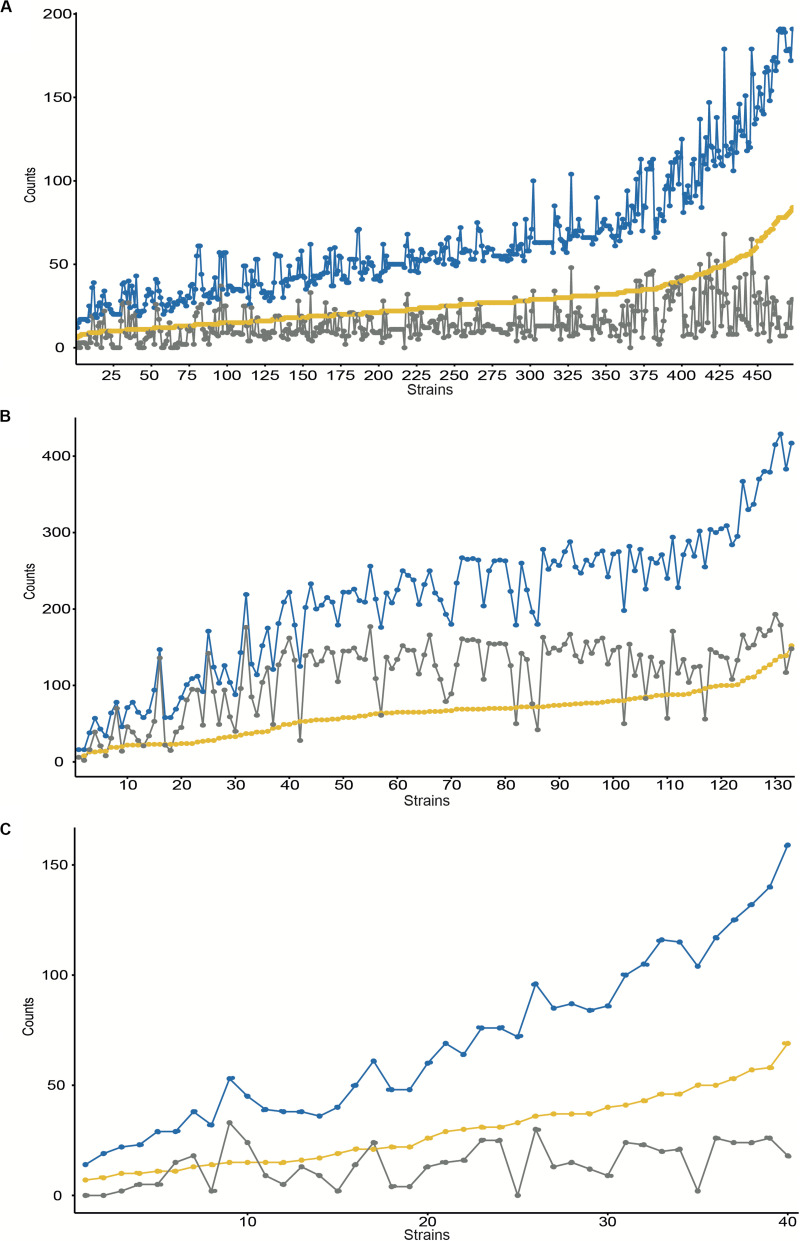
Number of gene duplications identified at the complete assembled genomes for NCBI Refseq entries for *S. aureus* **(A)**, *E. faecalis* **(B)**, and *E. faecium* **(C)**. The Y-axis contains the number of counts for the number of duplicated groups (yellow), the number of gene duplications (blue), and the duplicated transposases (gray). Results are ordered by duplicated groups in increasing order for each strain (axis X).

We searched first for the presence of duplications in the overall number of 473 *S. aureus* genomes available at the Refseq NCBI database (Supplementary Table S1) performing an all-vs.-all protein search by BLASTp for each strain (Figure 1A and Supplementary Table S4). We also looked for the presence of methicillin resistance determinants and the putative insertion of phages within each genome. From the 473 genomes analyzed, some contain more than 50 groups of duplicates, with up to 190 duplicates. Duplications range from 6 to 84 groups, with more than 50% of the strains encoding more than 26 groups of duplicates. No clear correlation was identified between the total number of duplicates and the number of duplicated transposases annotated (*R*^2^ = 0.332, pval-adj = 1.894e-43). On the other hand, a slight correlation was identified between the total number of duplicates and duplicated hypothetical or proteins of unknown function (*R*^2^ = 0.8, pval-adj = 4.735e-167 and *R*^2^ = 0.532, pval-adj = 7.439e-80, respectively). We also explored the distribution of duplicates under a cutoff of phages inserted. Those strains with >2 phages inserted contain, on average, more duplicates (pval = 0.024).

With regard to *E. faecium*, we searched for the presence of gene duplications within each strain in all 133 genomes available at the NCBI Refseq database (Supplementary Table S1) by using the same BLAST strategy described above (Figure 1B and Supplementary Table S5). From the 133 genomes analyzed some contain more than 50 groups of duplicates, with up to 429 duplicates. Duplications range from 6 to 152 groups, with more than 50% of the strains encoding more than 66 groups of duplicates. A half correlation was identified between the total number of duplicates and the number of duplicated transposases annotated (*R*^2^ = 0.708, pval-adj = 4.828e-37) and with the duplicated hypothetical proteins annotated (*R*^2^ = 0.721, pval-adj = 2.0439e-38). We also explored the distribution of duplicates under a cutoff of phages inserted. Those strains with >4 phages inserted contain, on average, more duplicates (p-val = 0.0017).

To complete our survey of duplications, we searched for the presence of duplications in all 40 *E. faecalis* genomes available (Supplementary Table S1) by using the same strategy described above (Figure 1C and Supplementary Table S6). From the 40 genomes analyzed, some contain more than 50 groups of duplicates, with up to 159 duplicates. Duplications range from 14 to 69 groups, with more than 50% of the strains encoding more than 26 groups of duplicates. No clear correlation was identified between the total number of duplicates and the number of duplicated transposases annotated (*R*^2^ = 0.320, pval-adj = 8.4656e-05). A medium correlation was identified with the duplicated hypothetical proteins annotated (*R*^2^ = 0.787, pval-adj = 1.435e-14). We also explored the distribution of duplicates under a cutoff of phages inserted. Those strains with >4 phages inserted contain, on average, more duplicates (p-val = 0.0014).

### Duplications in *S. aureus* Strain Newman

To further study the gene duplications in *S. aureus* genomes, we decided to analyze them in a well-characterized strain such as *S. aureus* Newman. It was isolated in 1952 from a human infection (Duthie and Lorenz, 1952) and has been commonly used as a model strain both for studying *S. aureus* pathogenesis (Richardson et al., 2008; Alonzo et al., 2013) and for the assessment of the therapeutic efficacy of antimicrobial compounds designed to threat *S. aureus* infections (Thammavongsa et al., 2013; Zhang et al., 2014). Its genome sequence has been available since 2008 (Baba et al., 2008).

**FIGURE 2:**
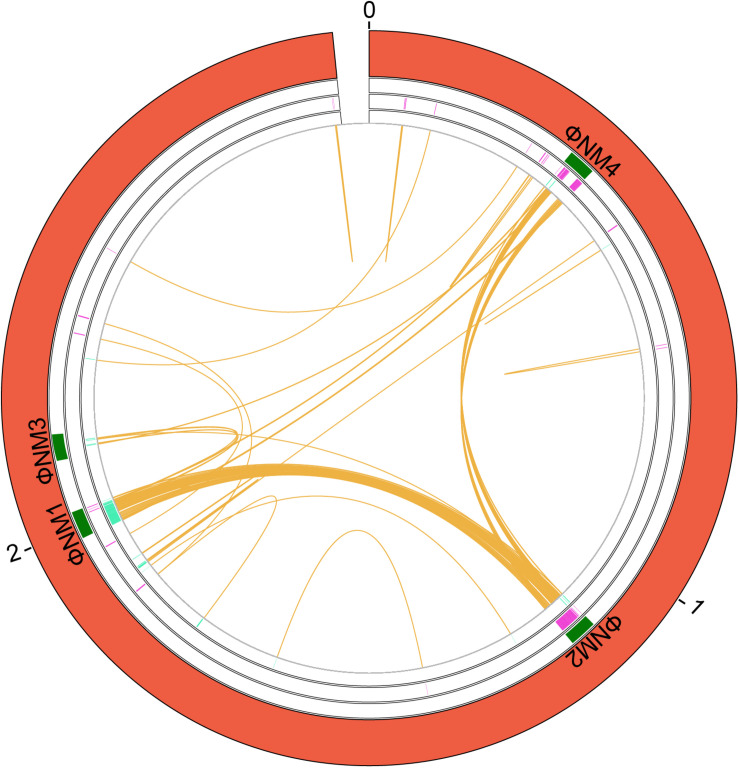
Genes duplicated in the *S. aureus* strain Newman. A circular map of the chromosome showing the duplications is shown. From the outside in, the outer circle represents the chromosome (red). The second circle includes the position of the identified phages that are inserted in the chromosome (green). Next circle shows the duplicated genes in the (+) strand at each coordinate (purple). The innermost circle shows the duplicated genes in the (−) strand at each coordinate (turquoise). The orange color shows the connection between duplicates. The size is shown in Mb.

We analyzed the extent of gene duplications in strain Newman (GCA_000010465.1), and mapped along the Newman genome those genes that are present in two or more copies (Figure 2 and Supplementary Table S7). A total number of 78 genes are duplicated in that strain. Most of the duplicated genes are located in two main regions (Supplementary Table S7). The insertion phage analysis identified five putative phages within the chromosome of this strain. Four of this phage coordinates match quite well with four prophages previously described in the Newman strain (Bae et al., 2006; Baba et al., 2008). Many of the genes that are duplicated in this strain are in the same coordinates as these phages (Figure 2). Specifically, several duplicates correspond to genes of phages ΦMN4, ΦMN2, and ΦMN1. Some ΦMN4 genes are present in both ΦMN2 and ΦMN1 and hence are present as triplicates. Other ΦMN2 genes are also present in ΦMN1, and are therefore present as duplicates (Supplementary Table S7). A small percentage of the duplicates maps outside the phage genomes (Figure 2).

With regard to the gene functions of the duplicated genes, 33% correspond to hypothetical proteins, 20% of proteins of unknown function, 19% to phage proteins, and the rest of proteins display miscellaneous functions (Supplementary Table S7).

### Duplicated Genes From Strain Newman That Are Also Duplicated in Other *S. aureus* Strains

**FIGURE 3:**
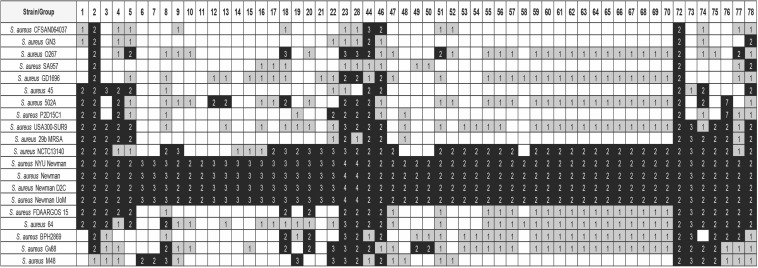
Distribution of some of the detected groups of duplicated genes in strain Newman, in 20 selected *S. aureus* strains. The white, gray, and black colors indicate, respectively, gene absent; gene present in a single copy; gene present in two or more copies. The numbers show the copy number of each gene. The order of the strains follows its phylogenetic relationship. Analysis of the whole set of 473 genomes is shown in Supplementary Table S8. Details of the duplicated groups are referred in Supplementary Table S7.

We addressed next the question as to whether the existing duplicates in this *S. aureus* strain are strain-specific or, on the contrary, they were generated in some putative ancestor and are also present in many other *S. aureus* strains. We used the 473 *S. aureus* genomes to check the shared duplicated genes (see Material and Methods for details). A representative summary of the results obtained is presented in Figure 3. The complete analysis is detailed in Supplementary Table S8.

As expected, other sequenced Newman strains [strains 412 (GCA_002310435.1), 414 (GCA_002310395.1), and 415 (GCA_900092595.1)] show the same duplication pattern than that obtained with the strain used for the analysis of duplications in the genome [strain 413 (GCA_000010465.1)]. It is also remarkable that strain NCTC13140 [strain 411 (GCA_900474725.1)] shows a gene duplication pattern quite similar to that of the Newman strain. This suggests a close phylogenetic relationship between this strain and the different Newman strains.

The analysis performed also shows that two of the duplicates in strain Newman are also duplicated in most of the strains analyzed (Table 1). These genes are *deoC* (group 2) and *rpmG* (group 72). Mutations in *deoC* (codes for the enzyme deoxyribose phosphate aldolase), *prkC* (codes for the serine/threonine-protein kinase PrkC), *plsY* (codes for the enzyme glycerol-3-phosphate acyltransferase), *eap* (codes for an extracellular adherence protein), and *sstD* (codes for an iron-binding protein belonging to an ABC uptake transporter) have been shown to result in the generation of small colony variants (SCV) of *S. aureus* (Chen et al., 2018). We analyzed therefore whether there also existed duplicates of other genes related to the formation of SCVs in all staphylococci (Supplementary Table S2). For this analysis, a lower similarity cutoff (>50%) was used in order to detect duplicates with lower similarity (Supplementary Table S9). Out of *deoC*, the rest of the genes that have been associated to the generation of SCVs are not duplicated in the genus *Staphylococcus.* Interestingly, *deoC* is duplicated in all *S. aureus* strains analyzed, but not in other catalase negative staphylococci.

**TABLE 1:** Details of the selected duplicated genes of the strain S*taphylococcus aureus* Newman that are also duplicated in other *S. aureus* strains.

Group	Locus tag 1	Symbol	Description	Percentage
1	NWMN_RS00250	*csa1A*	Tandem type lipoprotein	50.95%
2	NWMN_RS00465	*deoC*	Deoxyribose phosphate aldolase	98.94%
44	NWMN_RS02255	*hsdM*	Type I restriction-modification system subunit M	76.74%
72	NWMN_RS07040	*rpmG*	50S ribosomal protein L33	100.00%
74	NWMN_RS09560	*splF*	Serine protease	53.07%
75	NWMN_RS09635	*bsaA2*	Gallidermin/nisin family lantibiotic	36.79%
78	NWMN_RS14900	*vraH*	Peptide resistance ABC transporter activity modulator	37.00%

*Percentage of the analyzed strains that contain the duplicated gene is shown.*

The *rpmG* gene codes for the ribosomal protein L33. The existence of duplicates of the genes coding, among other ribosomal proteins, for the ribosomal protein L33 was already reported in some Gram-positive microorganisms (i.e., *Bacillus subtilis*, *B. anthracis*, and *Lactococcus lactis*) as well as in some mycoplasma (Makarova et al., 2001; R Development Core Team, 2008; Kandari et al., 2018).

Out of *deoC* and *rpmG*, another set of genes are duplicated in a group of about 250 strains that are phylogenetically related (Figure 3 and Supplementary Table S8). From these genes, (groups 1, 3, 4, 5, 8, 18–23, 28, 44, 46, 73–78; Supplementary Table S7) eight are phage genes (groups: 8, 18–23, 28). From the rest, the function of some is known (Table 1). They code, respectively, for a lipoprotein (group 1, gene csa1A), for the M subunit of a restriction/modification system (group44, gene *hsdM*), for the SplF serine protease (group 74, gene *splF*), for a lantibiotic of the gallidemin/nisin family (group 75, gene *bsaA2*), and for a gene belonging to the v*raDEH* operon (group 78, gene vraH), associated to the *S. aureus* resistance to antimicrobial peptides and to cells survival in an infection model (Popella et al., 2016). *S. aureus* Spl proteases are believed to induce allergic reactions (Stentzel et al., 2017).

### Duplications in *E. faecium* Strain 6E6

**FIGURE 4:**
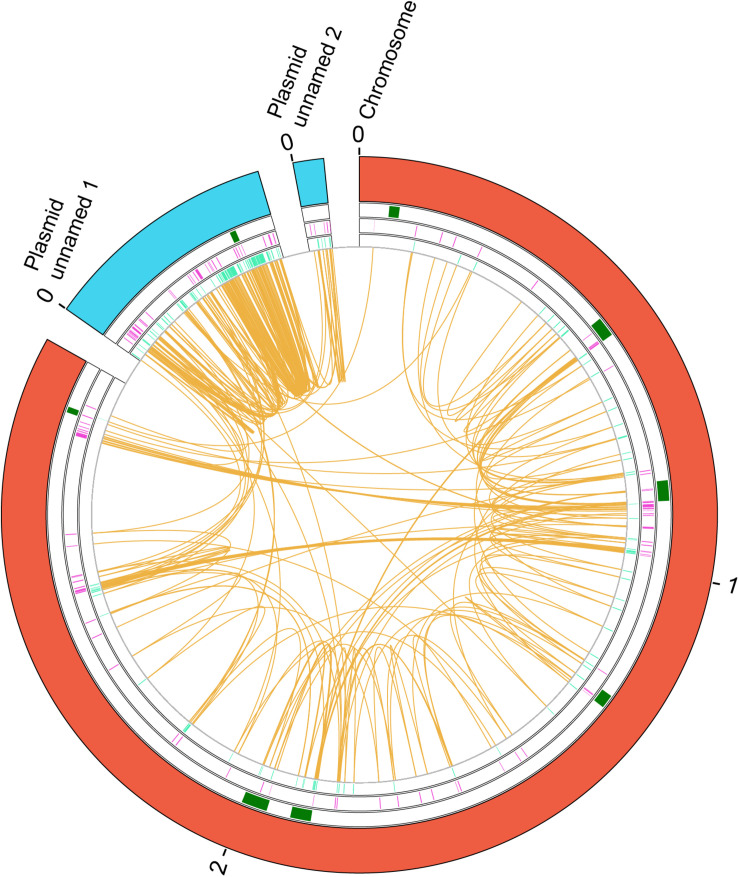
Genes duplicated in the *E. faecium* 6E6 strain. A circular map of the genome of the *E. faecium* 6E6 strain showing the duplicates in the chromosome and in two plasmids is shown. From the outside in, the outer circle represents the chromosome (red) and the plasmids (blue). The second circle includes the position of the identified phages that are inserted in the chromosome (green). Next circle shows the duplicated genes in the (+) strand at each coordinate (purple). The innermost circle shows the duplicated genes in the (−) strand at each coordinate (turquoise). The orange color shows the connection between duplicates. The size is shown in Mb.

We used for the study strain 6E6 (GCA_001518735.1), a vancomycin resistant isolate from the University of Minnesota (Geldart and Kaznessis, 2017), that contains a large number of duplicates (*n* = 337). We identified and mapped those genes that are present in two or more copies in strain 6E6 (see section “Materials and Methods” for details; Figure 4 and Supplementary Table S10). A total number of 111 gene groups are duplicated in that strain. Some of them (23%) correspond to transposases, which are present in three or more copies (Supplementary Table S10). Several of the duplicates contain at least one of the copies in a plasmid (Figure 4). With regard to the gene functions of the duplicated genes, 41% code for hypothetical proteins, 4.5% code for proteins of unknown function, and the rest for proteins with varying functions (Supplementary Table S10). The high percentage of duplicates that code for proteins of unknown function can be correlated with the HGT origin of these genes.

### Duplicated Genes From Strain 6E6 That Are Also Duplicated in Other *E. faecium* Strains

**FIGURE 5:**
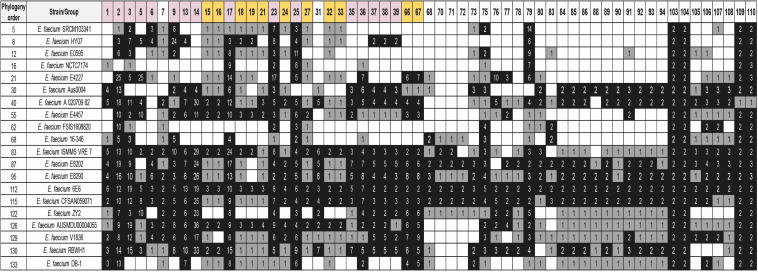
Distribution of some of the detected groups of duplicated genes of strain 6E6 in 20 selected *E. faecium* strains. BLASTp analysis was used for the study. The white, gray, and black colors indicate, respectively, gene absent; gene present in a single copy; gene present in two or more copies. Purple color corresponds to genes with at least one copy in the chromosome and one copy in a plasmid. The gold color corresponds to plasmid genes. The numbers show the copy number of each gene. The order of the strains follows its phylogenetic relationship. Analysis of the whole set of 133 genomes together with their phylogenetic relationship is shown in the Supplementary Table S11. Details of the duplicated groups are shown in Supplementary Table S10.

Once we determined the gene duplications in the *E. faecium* 6E6 genome, we analyzed whether the existing duplicates are also present in other *E. faecium* strains. We used the 133 *E. faecium* genomes to check the shared duplicated genes. Among the genes that are duplicated in strain 6E6 (Supplementary Table S10), a set of 26 genes are also duplicated in most if not all of the strains. A summary of representative data is presented in Figure 5 (see Supplementary Table S11 for the complete analysis). These duplicates can be divided in two groups. The first group (18 genes) includes either transposases or transposon-associated genes. At least one of the corresponding copies maps in a plasmid. The second group (eight genes) comprises four other genes that code either for phage proteins or for hypothetical proteins (groups 105–108), and four genes that code for proteins, which display well-characterized physiological functions (Table 2). They code, respectively, for a GlsB-like protein (group 103), for a LysM-like protein (group 104), and for two proteins of the PTS system: the lactose/cellobiose IIA and IIB subunits (groups 109 and 110, respectively).

**TABLE 2:** Details of the selected duplicated genes of the strain *Enterococcus faecium* 6E6 that are also duplicated in other *E. faecium* strains.

Group	Locus Tag 1	Description	Percentage
103	AWJ25_RS06350	GlsB/YeaQ/YmgE family stress response membrane protein	99.25%
104	AWJ25_RS07455	LysM peptidoglycan binding domain containing protein	99.25%
109	AWJ25_RS09645	PTS lactose/cellobiose transporter subunit IIA	95.49%
110	AWJ25_RS09650	PTS sugar transporter subunit IIB	78.20%

*Percentage of the analyzed strains that contain the duplicated gene is showed.*

### Duplications in the *E. faecalis* Strain V583

We selected for this study as a reference strain *E. faecalis* V583 (GCA_000007785.1), a VanB-type vancomycin-resistant virulent isolate that is a model strain for *E. faecalis* studies (Paulsen et al., 2003).

**FIGURE 6:**
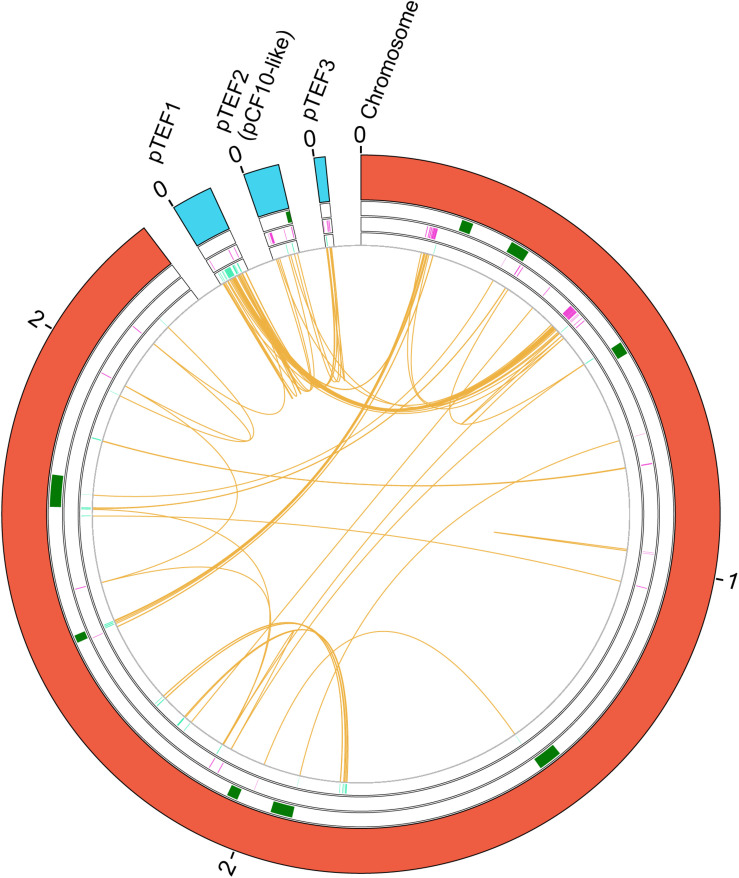
Genes duplicated in the *E. faecalis* V583 strain. A circular map of the genome showing the duplicates in the chromosome and in three plasmids is shown. From the outside in, the outer circle represents the chromosome (red) and plasmids (blue). The second circle shows the position of the identified phages that inserted in the chromosome (green). The next circle shows the duplicated genes in the (+) strand at each coordinate (purple). The innermost circle shows the duplicated genes in the (−) strand at each coordinate (turquoise). The orange color shows the connection between duplicates. The size is shown in Mb.

We analyzed the gene duplications in strain V583 (see Materials and Methods for details), and mapped along the V583 genome those genes that are present in two or more copies (Figure 6 and Supplementary Table S12). A total number of 52 gene groups are duplicated in that strain. Some of them (5.7%) correspond to transposases (Supplementary Table S12). As it happened with *E. faecium* strain 6E6, a large number of duplicates (50.9%) are plasmid genes. With regard to the gene functions of the duplicated genes, 18 duplicated genes code for hypothetical proteins (35%), and the rest code for proteins with different functions (Supplementary Table S12).

### Duplicated Genes From Strain V583 That Are Also Duplicated in Other *E. faecalis* Strains

**FIGURE 7:**
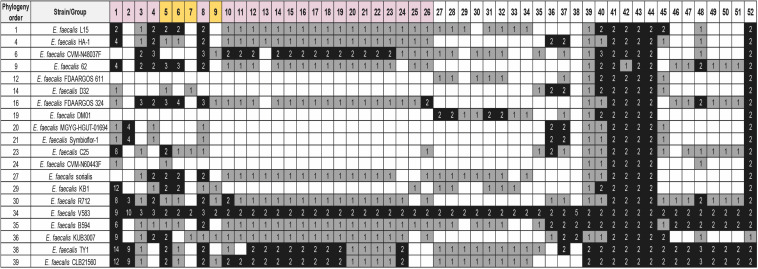
Distribution of the detected groups of duplicated genes of strain V583 in 20 selected *E. faecalis* strains. BLASTp analysis was used for the study. The white, gray, and black colors indicate, respectively, gene absent; gene present in a single copy; gene present in two or more copies. Purple color corresponds to genes with at least one copy in the chromosome and one copy in a plasmid. The gold color corresponds to genes with all copies in plasmids. The numbers show the copy number of each gene. The order of the strains follows its phylogenetic relationship. Analysis of the whole set of 40 genomes together with their phylogenetic relationship is shown in Supplementary Table S13. Details of the duplicated groups are shown in Supplementary Table S12.

We also analyzed if the existing duplicates in strain V583 are also present in many other *E. faecalis* strains. We used the 40 *E. faecalis* genomes (Supplementary Table S1) to check the shared duplicated genes with strain V583 (see Material and Methods for details) (Figure 7 and Supplementary Table S13). In contrast to *E. faecium*, only a small group of duplicates (five) is present in almost all the strains analyzed. All of them are chromosomal duplicates. The *dgaEF* genes (groups 41 and 42) are required for microbial growth on glucose aminoate (Miller et al., 2013). The genes from group 43 code for the subunit IIB of the PTS system. This gene is also duplicated in *E. faecium*. The genes from group 44 (*galE*) code for the UDP glucose 4 epimerase that catalyzes the last step of the Leloir pathway for the assimilation of galactose. The genes from Group 52 code for a protein containing a LPXTG cell wall anchor domain (Table 3).

**TABLE 3:** Details of the selected duplicated genes of the strain *Enterococcus faecalis* V583 that are also duplicated in other *E. faecalis* strains.

Group	Locus Tag 1	Symbol	Description	Percentage
4	EF_RS00670	*prgB*	LPXTG anchored aggregation substance	32.50%
8	EF_RS02380	*prgU*	Pheromone response system. RNA binding regulator	30.00%
21	EF_RS02400	*prgC*	Cell wall surface anchor family protein	7.50%
26	EF_RS00665	*sea1/prgA*	Surface exclusion protein	5.00%
37	EF_RS01780		LysM peptidoglycan binding domain containing protein	35.00%
39	EF_RS02105	*kduI*	5-dehydro-4-deoxy-D-glucuronate isomerase	17.50%
40	EF_RS03725	*cspA*	Cold shock protein	55.00%
41	EF_RS03995	*dgaE*	Pyridoxal phosphate dependent ammonia lyase family protein	100.00%
42	EF_RS04000	*dgaF*	KDGP aldolase family protein	97.50%
43	EF_RS04815	*celA*	PTS sugar transporter subunit IIB	100.00%
44	EF_RS05170	*galE*	UDP glucose-4-epimerase	100.00%

*Percentage of the analyzed strains that contain the duplicated gene is showed.*

Two other groups of duplicates are present in a significant number of the strains analyzed: groups 1–9, encoded in plasmids, and groups 36–40, encoded in the chromosome. From groups 1–9, it is relevant to mention here the *prgB* and *prgU* genes (groups 4 and 8, respectively). *E. faecalis* strains harboring plasmid pCF10 respond to the presence of the peptide pheromone cCF10 by expressing three surface adhesins: PrgA, PrgB, and PrgC. They play a relevant role in host tissue attachment and biofilm formation (Gilmore et al., 2014). Overexpression of PrgB can be highly toxic to *E. faecalis* cells, and PrgU mitigates toxicity by downregulating PrgB synthesis (Bhatty et al., 2017). It was already reported that strain V583 contains several copies of the *prgU* gene (Bhatty et al., 2017). We show here that this duplication is present in several other *E. faecalis* strains.

With respect to groups 36–40, they code, respectively, for a holin, for a protein containing a LysM peptide-binding domain, for a transposase, for a protein that participates in pectin degradation (the *kdu* gene product) and for the cold shock protein CspA (Table 3).

Strains V583, VE18379, VE14089, and VE18395 that appear to share a set of duplicates, are closely related. Strain VE14089 is plasmid-free V583. Strains VE18379 and VE18395 are derivatives from strain VE14089.

### Genomic Context of Some Core Genes That Are Duplicated in These Species

**FIGURE 8:**
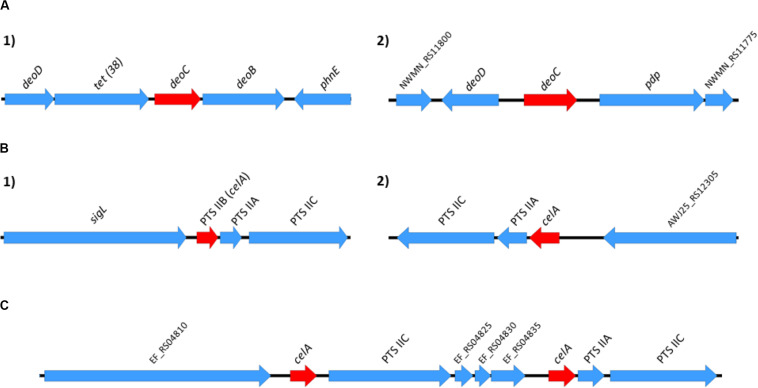
Genomic context of the *S. aureus deoC* duplicated genes **(A)** and of the *E. faecium* and *E. faecalis celA* duplicated genes (**B,C**, respectively). The genomic context was analyzed in the following representative strains: *S. aureus* Newman, *S. aureus* N315, *S. aureus* USA300, and *S. aureus* MW2; *E. faecium* 6E6, *E. faecium* Aus0004 and *E. faecium* DO; *E. faecalis* V583, *E. faecalis* OG1RF and *E. faecalis* Symbioflor. Red arrows in positions 1 and 2 correspond to the alleles of the duplicated genes. The genomic context for the *deoC* and *celA* genes was identical in all *S. aureus* and *E. faecium/E. faecalis* strains analyzed.

A relevant question to be addressed is whether the identified duplicated genes that code for core functions result either from ancient duplications and are located in fixed points of the chromosome, or have been generated because of some of these genes being flanked by IS elements and jumping to different positions in the chromosome. To assess this, we analyzed the genomic context of both copies of the *deoC* gene in representative strains of *S. aureus*, and of the *celA* gene in representative strains of *E. faecium* and E. *faecalis* (Figure 8). The genomic context of the two alleles of the *deoC* and *celA* genes is the same in the different strains analyzed. They are not surrounded by IS elements, but by other core genes.

## Discussion

It is apparent that in the Gram-positive cocci studied in this work, mobile DNA elements encode a significant part of the duplicates present in their genomes. The statistical analysis performed correlates duplicates both with phages inserted in the chromosome and with genes encoding either hypothetical or proteins of unknown function (much more common in mobile DNA than in the core genome). In *S. aureus*, most of the duplicated genes are of phage origin. This is not surprising because of the relevant role that phages have in the biology of this microorganism (Xia and Wolz, 2014; Ingmer et al., 2019). In contrast, plasmids are the predominant mobile elements, which encode a significant part of the duplicates in the enterococci. In *E. faecium* there exists a significant correlation between duplicates and transposases. This is also shown when the existing duplicates are identified by BLAST analysis. The question to be addressed is the biological significance of the duplication of genes encoded in mobile DNA in these microorganisms.

Although duplicates located in mobile DNA predominate in the microorganisms studied here, others are located in the chromosome. Some of these latter duplicates are widespread among all the strains of the same species analyzed. In *S. aureus*, two duplicates are present in almost all strains analyzed: the *rpmG* and the *deoC* genes. The former codes for the ribosomal protein L33. Duplications of the gene encoding the L33 protein appear to be a hallmark of several Gram-positive genera. As a general rule, one of the *rmpG* paralogs codes for a protein that contains a Zn-binding motif comprising a two pair of conserved CXXC stretch (CC form), which is absent in the other (C- form). In strain Newman, two copies are C- (NWMN_RS07040, and NWMN_RS08205, respectively), and the third is CC [NWMN_0496.1 (this latter shows less than 85% identity)]. In addition to their role in translation, ribosomes also serve as reservoirs for zinc in the cell (Moore and Helmann, 2005). The zinc-responsive regulator Zur has been shown to repress the C- form (Akanuma et al., 2006; Gabriel and Helmann, 2009). Under zinc-depleted conditions, the Zur mediated repression of the genes encoding the C- forms of the ribosomal proteins is alleviated. These C- forms then replace the corresponding CC forms from the ribosomes, resulting in exoneration of zinc, which can then be used by other metalloproteins. This enables the bacterial cell to survive in zinc limiting environments (Moore et al., 2005; Akanuma et al., 2006).

The *deoC* gene product is the deoxyribose phosphate aldolase, which enables bacterial cells to grow on deoxyribonucleosides as the carbon source. As commented above, mutations in the *S. aureus deoC* gene have been associated with the generation of SCVs (Chen et al., 2018). Interestingly, *deoC* mutations were associated with alterations in the response to extracellular signaling in *E. coli* (Joloba and Rather, 2003). It can be hypothesized that, as the *deoC* gene is duplicated in *S. aureus* but not in other catalase negative cocci, its gene product can play a role in *S. aureus* virulence.

In addition to these widespread duplicates, another group of duplicates is present in a subset of the *S. aureus* genomes. The strains containing these duplicates are phylogenetically related. The reported functions for these genes (i.e., *vraH*, *splF*) are also related to virulence, and they can be considered as virulence markers of that group of *S. aureus* strains. A question to be addressed is whether the duplication of these genes confers specific virulence features to *S. aureus*.

In *E. faecium* genomes, a significant part of the duplicates (58%) are located (at least one of the copies) in plasmids. Several of these duplicates code either for transposases or for hypothetical proteins. Some of them are shared by most of the strains analyzed. In addition to these genes of plasmid origin, four chromosomal genes are also duplicated in most of the *E. faecium* strains analyzed. Expression of GlsB proteins has been associated with virulence and bile salt stress (Choudhury et al., 2011; Zhang et al., 2013a). Proteins containing a LysM domain have been shown to be induced under infection conditions of a mammalian host (Cacaci et al., 2018). Although the proteins of the lactose/cellobiose PTS system IIA and IIB have not been hitherto described as relevant elements in *E. faecium* virulence, the relevance of the PTS system for the ability of *E. faecium* to colonize the host has been previously reported. Deletion of the *pstD* gene, which is predicted to encode the enzyme IID subunit of a PTS system, influenced *E. faecium* virulence (Zhang et al., 2013b). Furthermore, insertional inactivation of the *bepA* gene, coding for putative a PTS permease, was found to be relevant for *E. faecium* pathogenesis (Paganelli et al., 2016). The fact that, as shown in this report, other PTS specific components are duplicated in *E. faecium* strains further highlights the role of the phosphotransferase system in *E. faecium* physiology and hence, in the ability of virulent strains to colonize their hosts.

As it happens in *E. faecium*, about half of the *E. faecalis* duplicates are located (at least one of the copies) in a plasmid. Nevertheless, in contrast to *E. faecium*, few of the *E. faecalis* duplicates that are located in plasmids are transposases. A cluster of duplicated plasmid genes (*prgABCU*) are of special relevance. Its gene products enable *E. faecalis* to respond to the presence of the peptide pheromone cCF10 by expressing the surface adhesins PrgA, PrgB, and PrgC (Gilmore et al., 2014). It has been suggested that *prgU* expression controls *prgB* expression, avoiding that excess of the PrgB protein can be deleterious for the cell. *prgB* and *prgU* are present in several copies in strain V583, and there exists a genetic linkage between both genes (Bhatty et al., 2017). We show in this work that gene duplication occurs predominantly with both the *prgB* and *prgU* genes, and not with *prgA* and *prgC* genes. Our data are hence consistent with the genetic linkage of *prgB a*nd *prgU* (Bhatty et al., 2017). *prgU* genes are widely distributed on plasmids and chromosomes of *E. faecalis* and other enterococci, and it has been suggested that the *prgB-prgU* genetic linkage might have evolved to ensure the controlled synthesis of PrgB-like adhesins (Bhatty et al., 2017). Accordingly, we show here that the genetic linkage of *prgB-prgU* also involves gene duplication. In accordance with the rule that we suggested previously for *E. coli* (Bernabeu et al., 2019), the duplication of the regulated gene (*prgB*) correlates with the duplication of its modulator (*prgU*).

In *E. faecalis* there also exists a group of duplicates located in the chromosome that is shared by all the strains analyzed. Three of them code for proteins playing a role in cell metabolism, including the duplication of the gene coding for of the subunit IIB of the lactose/cellobiose PTS system. Different components of the PTS system have also been reported as relevant for *E. faecalis* colonization (Paulsen et al., 2003), and they have deserved special attention in the last years (Ruiz-Cruz et al., 2015; Sauvageot et al., 2017; Grand et al., 2019).

Both for *S. aureus* and *Enterococcus*, the genomic context of the duplicates coding for core genes is similar among strains, and corresponds to other core genes. This fact, together with the widespread distribution of these genes among all strains analyzed suggests that these duplications correspond to ancient events that have been positively selected in the course of evolution.

Genomic analysis is powerful to gain insight into several aspects of the biology of organisms. We show here that the analysis of the pattern of gene duplications in microorganisms can provide relevant information that can be useful for both establishing phylogenetic relationships between strains, and for the identification of genes that can play relevant roles in, among other processes, bacterial virulence.

## Data Availability Statement

All datasets generated for this study are included in the article/Supplementary Material.

## Author Contributions

JS-H, MB, and AJ designed this study. JS-H and MB performed the *in silico* work. JS-H, MB, AP, MH, and AJ wrote the manuscript. MH and AJ did the final version of the manuscript. All authors analyzed and discussed the results, read and approved the final manuscript.

## Conflict of Interest

The authors declare that the research was conducted in the absence of any commercial or financial relationships that could be construed as a potential conflict of interest.

## Footnotes

1 R-project.org

## Supplementary Material

TABLE S1 List of the complete assembled genomes corresponding to NCBI Refseq entries for *S. aureus*, *E. faecalis*, and *E. faecium* used for the gene duplication analysis.

TABLE S2 List of the NCBI Refseq entries for *S. epidermidis*, *S. carnosus*, and *S. xylosus* used for the analysis of the duplications of genes involved in the generation of small colony variants (SCV).

TABLE S3 Details of the genes involved in the generation of SCVs used for the duplication analysis.

TABLE S4 Gene duplication data obtained for the *S. aureus* strains analyzed. Indicated are: (i) Phylogeny order: according to the clusterization analysis using mash and sourmash tools. (ii) Groups: counts of groups of duplicated genes. (iii) Dups: total count of duplicated genes. (iv) Transpo: counts of genes within duplicates annotated as “transposase”. (v) Hypo: counts of gene names within duplications annotated or containing “hypothetical” functions. (vi) Phages: Number of putative phages identified with PhiSpy inserted in the genome. (vii) MRSA: presence of methicillin resistance determinants *mecA* and/or *mecC*. The order of the strains follows its phylogenetic relationship.

TABLE S5 Gene duplication data obtained for the *E. faecium* strains analyzed. Indicated are: (i) Phylogeny order: according to the clusterization analysis using mash and sourmash tools. (ii) Groups: counts of groups of duplicated genes. (iii) Dups: total count of duplicated genes. (iv) Transpo: counts of genes within duplicates annotated as “transposase”. (v) Hypo: counts of gene names within duplications annotated or containing “hypothetical” functions. (vi) Phages: Number of putative phages identified with PhiSpy inserted in the genome. (vii) VRE: presence of vancomycin resistance determinants *vanA* (blue), *vanB* (yellow), *vanA*/*vanB* (green), or *vanG* (red). The order of the strains follows its phylogenetic relationship.

TABLE S6 Gene duplication data obtained for the *E. faecalis* strains analyzed. Indicated are: (i) Phylogeny order: according to the clusterization analysis using mash and sourmash tools. (ii) Groups: counts of groups of duplicated genes. (iii) Dups: total count of duplicated genes. (iv) Transpo: counts of genes within duplicates annotated as “transposase”. (v) Hypo: counts of gene names within duplications annotated or containing “hypothetical” functions. (vi) Phages: Number of putative phages identified with PhiSpy inserted in the genome. (vii) VRE: presence of vancomycin resistance determinants *vanA* (blue) or *vanB* (yellow). The order of the strains follows its phylogenetic relationship.

TABLE S7 Locus tag and description of the duplicates identified in *S. aureus* strain Newman. Red, blue and green colors correspond to genes located in the ΦMN4, ΦMN2, and ΦMN1 phages, respectively.

TABLE S8 Distribution of the detected duplicated genes of strain Newman in the genomes of the other 472 *S. aureus* strains analyzed. BLASTp analysis was used for the study. The white, gray, and black colors indicate, respectively: gene absent; gene present in a single copy; gene present in two or more copies. The numbers show the copy number of each gene. The order of the strains follows its phylogenetic relationship.

TABLE S9 Analysis of the duplications of genes *deoC*, *sstD*, *plsY*, and *eap* in *S. aureus*, *S. epidermidis*, *S. carnosus*, and *S. xylosus*.

TABLE S10 Locus tag and description of the duplicates identified in *E. faecium* strain 6E6. The purple and gold colors correspond, respectively, to genes with at least one copy in chromosome and one in plasmids, and to genes with all copies in plasmids.

TABLE S11 Distribution of the detected duplicated genes of strain 6E6 in the genomes of the other 132 *E. faecium* strains analyzed. BLASTp analysis was used for the study. The white, gray, and black colors indicate, respectively: gene absent; gene present in a single copy; gene present in two or more copies. Purple color corresponds to genes with at least one copy in the chromosome and one copy in a plasmid. The gold color corresponds to genes with all copies in plasmids. The numbers show the copy number of each gene. The order of the strains follows its phylogenetic relationship.

TABLE S12 Locus tag and description of the duplicates identified in *E. faecalis* strain V583. The purple and gold colors correspond, respectively, to genes with at least one copy in chromosome and one in plasmids, and to genes with all copies in plasmids.

TABLE S13 Distribution of the detected duplicated genes of strain V583 in the genomes of the other 39 *E. faecalis* strains analyzed. BLASTp analysis was used for the study. The white, gray, and black colors indicate, respectively: gene absent; gene present in a single copy; gene present in two or more copies. Purple color corresponds to genes with at least one copy in the chromosome and one copy in a plasmid. The gold color corresponds to genes with all copies in plasmids. The numbers show the copy number of each gene. The order of the strains follows its phylogenetic relationship.
